# Real-Time Mobile Teleophthalmology for the Detection of Eye Disease in Minorities and Low Socioeconomics At-Risk Populations

**DOI:** 10.1097/APO.0000000000000416

**Published:** 2021

**Authors:** Lama A. Al-Aswad, Cansu Yuksel Elgin, Vipul Patel, Deborah Popplewell, Kalashree Gopal, Dan Gong, Zach Thomas, Devon Joiner, Cha-Kai Chu, Stephen Walters, Maya Ramachandran, Rahul Kapoor, Maribel Rodriguez, Jennifer Alcantara-Castillo, Gladys E. Maestre, Joseph H. Lee, Golnaz Moazami

**Affiliations:** * New York University (NYU) Grossman school of Medicine, NYU Langone Health, NY, US; † Columbia University Medical Center, New York, NY, US; ‡ Department of Neuroscience, Department of Human Genetics, University of Texas Rio Grande Valley School of Medicine, Brownsville, TX, US

**Keywords:** access to care, diabetes and hypertension, leading causes of blindness, synchronies video consultation, teleophthalmology

## Abstract

**Purpose:**

To examine the benefits and feasibility of a mobile, real-time, community-based, teleophthalmology program for detecting eye diseases in the New York metro area.

**Design:**

Single site, nonrandomized, cross-sectional, teleophthalmologic study.

**Methods:**

Participants underwent a comprehensive evaluation in a Wi-Fi–equipped teleophthalmology mobile unit. The evaluation consisted of a basic anamnesis with a questionnaire form, brief systemic evaluations and an ophthalmologic evaluation that included visual field, intraocular pressure, pachymetry, anterior segment optical coherence tomography, posterior segment optical coherence tomography, and nonmydriatic fundus photography. The results were evaluated in real-time and follow-up calls were scheduled to complete a secondary questionnaire form. Risk factors were calculated for different types of ophthalmological referrals.

**Results:**

A total of 957 participants were screened. Out of 458 (48%) participants that have been referred, 305 (32%) had glaucoma, 136 (14%) had narrow-angle, 124 (13%) had cataract, 29 had (3%) diabetic retinopathy, 9 (1%) had macular degeneration, and 97 (10%) had other eye disease findings. Significant risk factors for ophthalmological referral consisted of older age, history of high blood pressure, diabetes mellitus, Hemoglobin Alc measurement of ≥6.5, and stage 2 hypertension. As for the ocular parameters, all but central corneal thickness were found to be significant, including having an intraocular pressure >21 mm Hg, vertical cup-to-disc ratio ≥0.5, visual field abnormalities, and retinal nerve fiber layer thinning.

**Conclusions:**

Mobile, real-time teleophthalmology is both workable and effective in increasing access to care and identifying the most common causes of blindness and their risk factors.

Rapid developments of telecommunication and information technology in the last decade brought along a rising field of medicine, that is, telehealth/telemedicine.^1^ Ophthalmology is a specialty that lends itself well to the implementation of telemedicine because interpretations of screenings are routinely used for diagnosis and prognosis of several eye diseases.^2^ Even though teleophthalmology began to develop in rural and remote areas with the idea of facilitating health care services for underserved populations, there is also a great need for it in both urban communities and high-income regions.^3’4^ The COVID-19 pandemic has showed how much essential it is.^5^ Although teleophthalmology is already accepted within the medical community to improve eye care adherence and access, there is still a great need for a teleophthalmologic protocol to detect the most common eye diseases.

Although the field of eye care remains stable despite its shortage of specialists, the growing population and the rise of old-age population leads to an exponential increase in follow-up visits, which indicates that the demand for teleophthalmology will continue to increase in the near future.^6,7^ We believe that an effective, economic, and comfortable screening way of teleophthalmologic approach will be an inevitable means to reduce hospital visits.

The objective of this pilot study is to examine the benefit and feasibility of a real-time mobile teleophthalmology program for screening of undetected eye diseases in the at-risk neighborhoods of the metro area and provide an evaluation for this program. In this program, vision test results were evaluated in real time and remote consultation with an eye care professional was immediately available. Our team previously conducted urban-located, community-based screenings with teleophthalmologic models to better understand different systematic approaches.^8,9^ In the current study, a real-time mobile teleophthalmology program was designed in the northern Manhattan of New York City. In a previous study using data from surrounding neighborhoods of northern Manhattan, both at fixed sites and in a mobile unit, it is shown that 25% of 8547 participants screened were glaucoma suspects, 15% were deemed to need further investigation of ocular diseases other than glaucoma, and 57% had never seen an eye doctor in their lifetime.^8,10^ These rates are highly motivating for further teleophthalmologic studies, including the current paper, to detect public health originated problems and modeling ideal teleophthalmologic modalities which, we believe, constitute an emergent need. For the current study, we hypothesized that people living in northern Manhattan were at risk of undiagnosed eye disease and that it would be worthwhile to make vision-evaluating services more readily accessible to them.

## METHODS

This study adhered to the tenets of the Declaration of Helsinki and was approved by the Columbia University Institutional Review Board.

**FIGURE 1.**
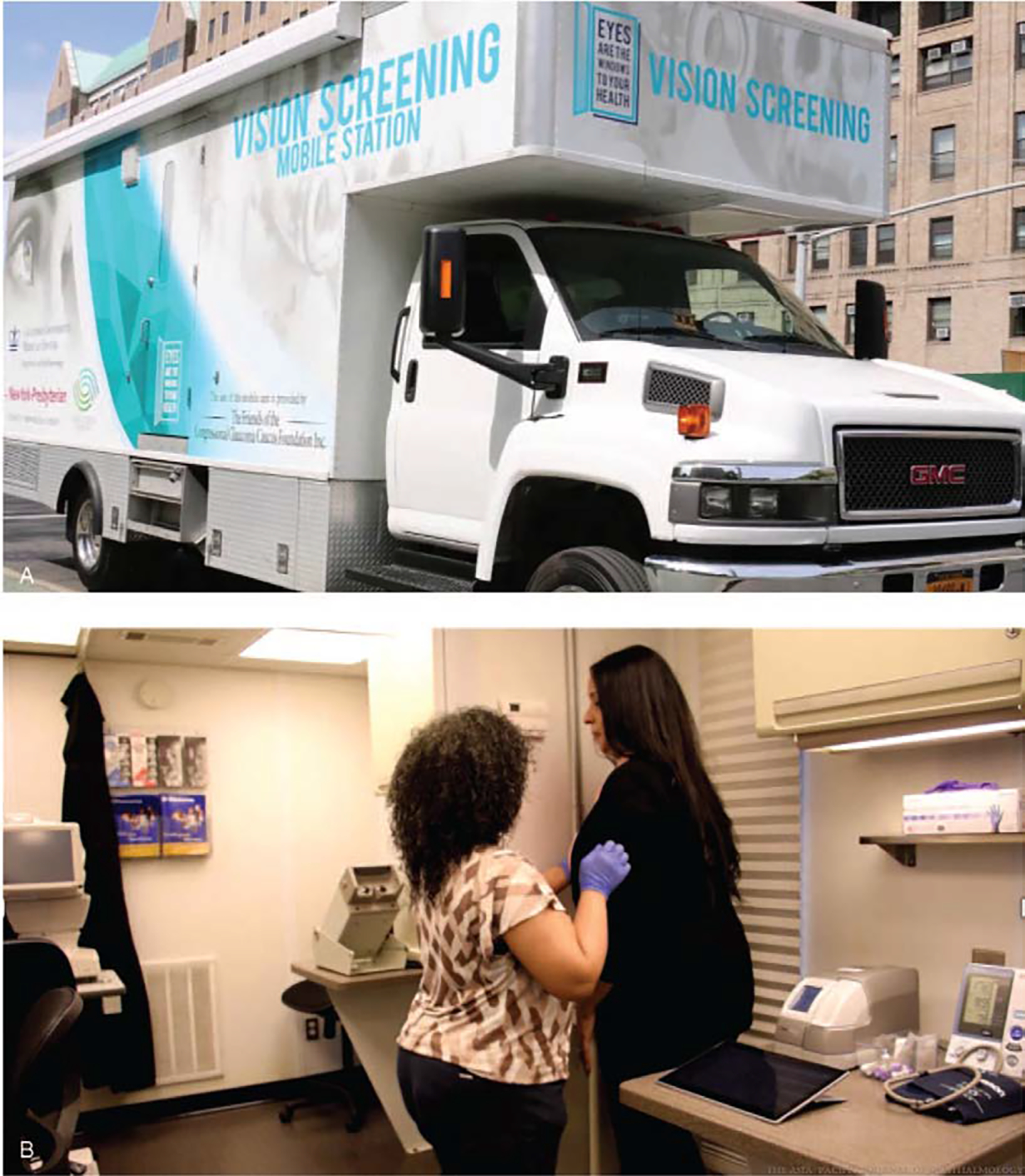
A, Teleophthalmology mobile unit. B, Inside of the mobile van.

Between June 2017 and November 2018, a Wi-Fi–equipped teleophthalmology mobile unit toured neighborhoods in the metro area (Fig. 1A). In some cases, the mobile unit was simply parked on a street; in other cases, it was parked at a community center or health fair. Flyers/handouts were distributed to announce the scheduled time and location for free vision screening. No other forms of recruitment were used. Subjects were not compensated for participating in the study. The only inclusion criterion for the study was to be 18 years of age or older.

In an isolated part of the mobile unit or hospital-affiliated screening center, an explanation of the study was provided in English to each prospective participant on a computer screen (or in hard copy, if desired) and the information was also given verbally. Alternatively, the explanation was available in Spanish, and Spanish-speaking staff members were present in the mobile unit to explain further if necessary. Participants clicked an “I Agree” button on the computer to indicate their willingness to participate, and this action generated a unique identification number. Three staff members including ocular technicians and medical and college students screened each participant as follows:

- Brief medical history taking that included self-identification of ethnicity and race, address of residence, past medical, ocular, and family histories including specific questions about diabetes mellitus, hypertension, sleep apnea, hyperlipidemia, smoking and dental examinations. This questionnaire was created to help the evaluation of risk factors for major eye diseases.

After this medical history taking process, participants were evaluated with basic systemic measurements such as:

- Height and weight measurement for calculation of body mass index (BMI)
- Blood pressure measurement using an electronic sphygmomanometer
- Hemoglobin Ale (HbAlc) testing (Alere Afinion point-of-care assay, Abbott, Abbott Park, IL)

Next, participants were taken to different stations for various ocular screening:

- Visual acuity measurement using the Snellen chart (Titmus 2s Vision Screener)
- Intraocular pressure (IOP) measurement via noncontact tonometry (Reichert 7CR Auto Tonometer, Cal Coast Ophthalmic Instruments Inc., Torrance, CA)
- Anterior and posterior segment optical coherence tomography (OCT) (3D OCT-1 Maestro, Topcon Medical Systems Inc., Oakland, NJ)
- Nonmydriatic Hindus photography (3D OCT-1 Maestro, Topcon Medical Systems Inc., Oakland, NJ)
- Peripheral visual field measurement using frequency doubling technology

The data obtained was entered and transmitted via a secure Virtual Private Network connection to the Edward Harkness Eye Institute reading center. The reading center is a centralized center where all data is securely transmitted utilizing an internally built data capturing system, imaging system, and video conferencing system. An ophthalmologist or optometrist at the center analyzed the data in real time. The eye care professional then video-conferenced in real time with the participant via Skype for Business, which incorporates end-to-end encryption for voice and video, to give recommendations for follow-up care. The conversation was conducted in a private part of the mobile unit. Before leaving the screening site, each participant received a printed copy of his or her results and recommendations for follow-up care as well as a copy of the informed consent form. Each participant also received a list of eye care professionals whose offices were in or near their neighborhood. All the evaluations including anamnesis, basic systematic measurements, ocular screenings, real-time video conference with an eye care professional and information about follow-up recommendations, took about 20 minutes for each participant. All the evaluations were performed in the efficient design mobile van (Fig. 1B) and only one participant was taken into the mobile van at a time to establish effective confidentiality.

To check follow-up rates and results within 2 to 4 months of the screening, participants who were referred to an ophthalmic examination were contacted by a patient navigator to ensure a follow-up visit with a local eye doctor. If participants failed to follow up, we inquired as to what prevented them from a follow-up to better understand the needs of the population.

The reading guidelines were prepared by an experienced glaucoma specialist for an interpretation of the findings. An experienced ophthalmologist or an optometrist analyzed the data in real time and referred to participants according to defined criteria.

### Definitions

#### Glaucoma suspect:

- IOP >21 mm Hg with corneal thickness taken into consideration, and/or glaucomatous appearance of the optic disc and/or an abnormal OCT consistent with glaucoma [deterioration of double hump appearance, asymmetry between 2 eyes in retinal nerve fiber layer (RNFL), generalized thinning in RNFL] and/or a narrow angle on anterior segment OCT and/or frequency doubling technology (FDT) abnormalities inconsistent with retinal pathologies.

#### Narrow-angle suspect:

- Structurally assessed angles based on anterior segment OCT. Angles were defined as narrow if they were ≤20 degrees on anterior segment OCT. The narrow-angle suspect group is categorized as a glaucoma suspect as well.

#### Cataract suspect:

- Visual acuity ≤20/40 with evidence of cataract on anterior segment OCT

#### Diabetic retinopathy:

- Hemorrhages or exudates on 45∞ fundus photography

#### Diabetic state:

- Prediabetic state: HbAlc between 5.7% and 6.4%
- Diabetes: HbAlc 6.5% or above^11^

#### Blood pressure guidelines:

- Low blood pressure: Systolic ≤90 mm Hg or diastolic ≤60 mm Hg
- Normal: < 120/80 mm Hg
- Elevated: Systolic 120–129 mm Hg and diastolic <80 mm Hg
- Stage 1 hypertension: Systolic 130–139 mm Hg or diastolic 80–89 mm Hg
- Stage 2 hypertension: Systolic ≥140 mm Hg or diastolic ≥90 mm Hg ^12^

#### BMI state:

- Obesity: BMI ≥30
- Above morbid obesity: BMI >40

The usual equation for calculating BMI (weight in pounds / height in inches^2^) was adjusted to account for the fact that the participants were clothed (–2.65 for males; – 1.76 for females).^13^

### Data Analysis

Relative risk ratios (RRR) for each predictor and their corresponding p-values were used for statistical inference. A multivariate Bayesian logistic regression was performed to identify patient characteristics associated with possible abnormal ophthalmic findings. A Bayesian approach to logistic regression was chosen to specify the prior distribution of each continuous variable and to avoid model overfitting. The regression model was built from the full set of candidate predictor variables, that is, all of the patient demographics and characteristics, and their pairwise interactions. Computerized statistical analyses were conducted using STATA software (version 14, StataCorp, College Station, TX). The alpha level (type 1 error) was set to be 0.05.

## RESULTS

Of 957 adults screened with 3828 total images, 3744 (97.81%) were readable images. No participant who wished to be screened was turned away, and no one refused screening once the study was explained. We eliminated 9 participants from the analysis because they did not finish the screening, or did not have images taken (fundus photographs and OCT images).

The median age of the participants was 58 years and 54% were female as shown in Table 1. The vast majority (93%) were ethnic/racial minorities (nonwhite Hispanic, 46%; African American, 31%; Asian, 10%; Caucasian, 6%; others, 7%). Evidently, the non-Hispanic and non–African American percentages in our study group are relatively small. That is why even though the large size of our study group would still allow some (but not strong) statistical comparison across different ethnicities, we refrain from doing so in the paper. Nevertheless, we should yield that this is a limitation for our study. Sizeable percentages had other risk factors for eye disease; close to one-third of the participants reported dyslipidemia, close to one-fifth were current smokers, and about 10% reported sleep apnea, which is a risk factor for glaucoma.^14^ One-third of the participants had not had a dental examination within 2 years (more than 5 years, 11%; never, 1%) and nearly half (43%) had not had an eye examination within 1 year (more than 5 years, 18%; never, 6%).

Of the 957 participants whose data was analyzed, 380 participants (40%) were newly diagnosed and 458 (48%) were referred for further ophthalmic evaluation. Of those, 305 (52%) were glaucoma suspects, 124 (25%) cataracts, 29 (6%) diabetic retinopathy, and 9 (1.8%) participants were macular degeneration suspects.

Since this was a community-based screening study, the only inclusion criterion for the study was to be 18 years of age or older. All participants who may have an ocular disease were evaluated with the same screening protocol as those who do not. Although some of the participants had self-reported eye conditions or were being monitored for a previously detected eye condition and disease, the newly identified disease rate is quite high as shown in Table 2; 261 of the glaucoma and 94 of the cataract suspects were referred to an ophthalmic examination with a novel prediagnosis. In addition, from the mobile screening 244 (25%) participants learned that they had diabetes or prediabetes and 183 (19%) of the study participants learned that they were hypertensive.

Table 3 shows various systemic examination findings among study participants, including HbA1c, systolic and diastolic blood pressure, and BMI measurements. As expected, the highest value of HbA1c, systolic blood pressure, and BMI were measured in the diabetic retinopathy group.

Table 4 shows various ophthalmologic parameters in study participants, including visual acuity (VA), intraocular pressure (IOP), central corneal thickness (CCT), vertical cup-to-disc ratio (VCDR, measured by reader and OCT), RNFL thickness, and FDT visual field (FDT-VF). Compared to those without any eye disease, the mean IOP was higher in patients with suspected glaucoma (14.56 mm Hg vs 16.89 mm Hg for the right eye, *P* value = 0.000, similar for the left eye). The mean CCT in patients with suspected glaucoma was similar to the mean CCT in healthy individuals (*P* = 0.55). The mean RNFL was notably thinner in patients with suspected glaucoma compared to the mean of the routine follow-up cohort (97.71 vs 108.58 for the left eye, P = 0.000, similar for the right eye). The cup-to-disc ratio in glaucoma suspects was larger on average by 0.13 mm compared to the healthy individuals (P = 0.00 for both eyes). Overall, and in all subgroups of participants by eye condition, the cup-to-disc ratio was greater when assessed by OCT than when assessed by the readers.

In Table 5, the effect of all the ophthalmologic parameters was evaluated using an RRR calculation for 5 different groups. Besides the demographic characteristics, the risk factors were grouped in 3 categories as: self-reported conditions, systemic findings, and ocular findings. These risk factors were further subdivided to 5 groups, ie, the groups with healthy ocular findings, and patients referred for a further ophthalmic evaluation, glaucoma, narrow-angle, and cataract suspects.

For the participants, age older than 65, personal history of hypertension, diabetes mellitus, glaucoma and cataract, presence of regular eye doctor, reported eye injury/surgery, a HbAlc measurement of 6.5, stage 2 hypertension, and as for the ocular parameters, all but CCT; including an IOP ≥21 mm Hg, VCDR ≥0.5, VF abnormalities, and RNFL thinning, negatively impacted the health status. All these parameters are associated with an increase in the incidence of ocular finding.

Not surprisingly, these parameters are all associated with a higher risk of being referred for a further ophthalmic evaluation, as well as for being classified as a glaucoma suspect. Interestingly, narrow-angle suspects who include the participants who have anatomically narrow angle at 3 and 9 o’clock on anterior segment OCT, have the only significant risk factors as age and female gender. None of the ocular findings, systemic findings, or self-reported conditions were significant.

Significant factors associated with a higher risk for cataract suspicion include age, personal history of hypertension-glaucoma-cataract, reported vision change, a HbAlc measurement of ≥5.7, both low and high blood pressure measurement, VCDR ≥0.5 and RNFL thinning. However, ethnic groups relative to Caucasians were found to be less at risk for cataract.

Table 6 presents the results of the Bayesian logistic regressions. The first 3 columns of Table 6 show the results of the regressions of being referred for further evaluation and the remaining 3 columns do the same for glaucoma suspicion. According to the presented results, aging, HbA1c, being previously treated for glaucoma, high IOP and low RNFL are all associated with a higher probability of being referred. The same factors are also significant for glaucoma suspicion.

As shown in Table 7, telephone follow-up was successful for only 38% of participants with cataracts, 38% with glaucoma, 30% narrow angles, 48% with retinal disorders, and none with macular degeneration. Besides, detected additional eye problems and disease confirmation rates were shown in Table 7.

## DISCUSSION

Our data indicate that 380 participants (40%) were newly diagnosed. 458 (48%) participants were detected for having at least 1 eye condition requiring treatment or control by an eye care specialist. It is worth emphasizing that this is not in an underserved neighborhood. Our team’s previous studies that were done in the same area showed that this ratio is 40.79% for an abnormal eye condition,^9^ and in another study, the percentage of the patients who were referred just for glaucoma evaluation was 25%.^8^ These rates were mainly described only as anatomically based abnormalities detected by comprehensive screening models and IOP measurements and perimetry. One should yield that peripheral retinal pathologies, corneal - lenticular - anterior segment pathologies require biomicroscopic evaluations, and therefore might be easily missed.

In this study, both the prevalence of detected ophthalmologic pathologies (48%) and newly diagnosed ocular conditions (40%) are absolutely higher compared to the results of similar studies. The prevalence of asymptomatic, newly diagnosed eye disease is 14% to 33% of patients in a great majority of the studies.^15–18^ It must be emphasized that, these studies are mainly retrospective analyses of optometry clinic documentations, 3 of which included subjects originating from spectacle prescription applications^15–17^ and 1 included patients who were regular users of general medical services.^18^ The only teleophthalmologic study that aimed to detect abnormal ocular conditions is the study of Grau et al in occupational medicine,^19^ but in this study, only 13.47% of the workers examined, whose ocular findings necessitating treatment or control by an ophthalmologist, were performed. Different from ours, this was based on an occupational medical study in Germany with a strikingly different population than ours and including insured subjects from working age population who are not largely coming from different ethnic origins contrary to our study.

The highest prevalence of abnormal ocular condition in literature is seen in our study. There might be several reasons that might contribute to this high rate. The high-risk populations in our subject pool have inadequate health insurance, irregular eye care, and ethnic and systemic risk factors. The participants screened for eye diseases in this pilot study, of whom 93% are racial or ethnic minority group, were confirmed to be considered underserved. 23% of these individuals did not have health insurance and more than half of them (57%) had not had an eye examination within the past year, including 18% who could not recall an eye examination within the past 5 years. An additional 6% said they had never had an eye examination. A similar report was published by Wang et al,^18^ who found that a substantial portion of the primary care clinic population (50%) in an urban community had undiagnosed ocular diseases and concluded that regular ophthalmic screenings would be required especially for patients who were over 65 years, were in poor health, had not had routine vision exams, or who did not have adequate insurance coverage. Their infrequent eye examinations, inadequate insurance coverage for eye care, and poor general health were considered to be relevant factors for the detection of ocular diseases. Another study, the Los Angeles Latino Eye Study, is yet another population-based study to detect undiagnosed eye diseases among Latinos finding out that any type of eye disease or refractive error frequency to be 53% in a population of older than 40.^20^ Ethnicity, education, lack of insurance, insurance coverage, lack of regular eye examination, and comorbidities were found to be significant factors in this regard. Therefore, there is a need to develop better strategies to educate the population about the importance of vision screenings and management of ocular diseases, especially as our population ages and the prevalence of diseases increases in the near future. This type of study can also help plan targeted educational awareness-raising campaigns to improve public health.

Another possible reason for the high rate of detection of the abnormal ocular conditions might be the comprehensiveness of our screening. Although a comprehensive screening generally assists clinicians in catching asymptomatic, mild diseases, it can also lead to false positivits.^21,22^ We modeled the screening protocol of our study using comprehensive tests as an aid, to prediagnose and to refer effectively to a definite examination. We used “either positive rule” for glaucoma references (the major part of the referenced group) if either structural test, functional test, or IOP was outside normal limits. Unexpectedly, more than one-third of participants had these criteria and were referred to a further ophthalmic examination as a glaucoma suspect. Large-scale population-based glaucoma screening studies on 40 years and older showed that the prevalence of patients referred for additional testing and ophthalmic examination is between 10%-33%^23–25^ and this is also less than our rate. These studies were not performed with a teleophthalmologic approach. Also, they did not include detailed anterior and posterior segment OCT screening for the RNFL and macula to catch early preperimetric glaucoma and narrow-angle glaucoma suspects.

In our study, glaucoma suspects are the largest group among all referrals. Similar to our study, the largest referred group (38.7%) in Maa et al^26^ is made up of glaucoma suspects. A high rate of glaucoma referral, complicated with asymptomatic early stages of glaucoma, led Maa et al to further their research with the next parts of their Technology-based Eyecare Services (TECS) study to establish consensus upon diagnosis. The TECS protocol was powered for glaucoma/glaucoma suspect detection at the first part^27^ and impact of OCT on the accuracy of the TECS protocol at the second part.^28^ These teleophthalmologic approaches prompted us to keep the definition criteria as sensitive as possible and to construct an OCT integrated model.

We used glaucoma suspect as an umbrella term to include all glaucoma possibilities (probable-definite, possible, likely) in a group of healthy participants who face risk factors for glaucoma (IOP >21mm Hg, narrow angle on anterior segment OCT, etc) or mimic glaucomatous appearance. Diagnosing glaucoma still remains a challenge as there is no single litmus test that can reliably tell whether glaucomatous changes are present. Even in the clinic, a definitive examination and the diagnosis of glaucoma is multifactorial and includes IOP, CCT, VF, disc appearance, OCT-RNFL findings, all of which were performed in this study. Despite all these factors, we should also yield that there is no single and definite consensus on glaucoma diagnosis parameters. Luckily, for most patients, these definitive parameters make the condition obvious that they either have or do not have glaucoma and they display objective documentation of the disease status. However, the detection of early glaucoma still remains challenging, as there is a significant overlap between normal variants and factors leading to an early detection of disease. Therefore, multiple diagnostic tests may play a massive role to overcome uncertainty. On the other hand, healthy subjects may still be categorized as glaucoma suspects due to “statistical abnormality” of outputs of diagnostic devices. Clinician affinity and over-reliance on newer diagnostic devices may lead to over-diagnosing glaucoma if findings are interpreted in isolation without taking into consideration the complete clinical scenario.^22,29^ Previous studies have also shown that the diagnostic accuracies of screening tests can vary with the severity of the disease and that the performance of the tests tail off from advanced to mild stage.^30–32^ The accuracy of tests may also vary according to the population characteristics.^29^ However, one should keep in mind that these previous studies were based at clinics, unlike our randomly sampled, population screening teleophthalmolgic model. This difference is especially important when one considers Maul and Jampel^33^ who argue a diagnostic test will not perform as well in the real world, even more so in a random sample of a population, as in a clinic setting. In line with this, Grødum et al^34^ showed that normaltension glaucoma, unilateral glaucoma, and better visual fields were more common in random populations compared to routine clinical glaucoma practice. This may suggest that even mild diseases are easily be missed in the population.

In summary, the inability to clearly distinguish between early glaucoma and normal variants is one of the major issues in glaucoma and it has persisted despite technological improvements, including teleophthalmologic innovations that may further aggravate the problem.

In the modeling phase of our study, definition criteria and reference conditions were aimed to be as sensitive as possible to cover all possible diseases. As the prevalence of open-angle glaucoma among adults in the US was stated to be 1.8% over the age of 40,^35^ this nonpractically high rate showed us the diagnostic tests “abnormality” rate in a more general population. In this regard, our study is an indicator of how large the positive screening findings might be in the population.

False positives generate direct costs due to unneeded further clinical examinations and excessive testing which may affect the participants’ quality of life. Even in face-to-face examination, it is possible to come across the unneeded initiation of treatment due to the slightest hint in ophthalmic screening. Therefore, mass ophthalmic screening and over-reliance of these techniques in teleophthalmology might lead to overtreatment, unnecessary medication costs, follow-up visits, and the risks of side effects without much gain. We believe the problems related to underdiagnosis and overtreatment can be tackled by creating a proper, well-designed teleophthalmologic model. For our future studies, we plan to continuously modify our algorithm based on lesson learned to decrease the false positive to create more accurate references.

Danish teleophthalmology platform is a good example to overcome this challenge.^36^ It was a real-world, large-scale, e-health based teleophthalmologic model. The authors of this study point out the requirement of an e-health model due to the dramatically risen eye care patients and concomitant referral system emergency. Their strategy was established to lighten the burden of reference system and it was mainly designed with a risk-stratified approach. According to the findings they lead the patients to an optometrist, telemedical service, or National Danish eye care service in the appropriate timing in one of the acute, subacute, and nonacute categories. Observing the group of patients with borderline or subacute findings in the telemedical service before referring them to the ophthalmology clinic is a well-thought model that optimizes the health care source. This would be an inspirational model for us in a future study.

Undoubtedly, devices cannot diagnose the patients and each condition requires a comprehensive assessment of personal history, risk factors, examination, and screening findings. In traditional clinical practice, information gained from the examination leads to ordering diagnostic tests where the clinician can then decide the probability of disease being present. The study we designed with this teleophthalmologic model is quite the opposite, beginning with a comprehensive screening which then leads to face-to-face examination for definite diagnosis. Despite the existence of potential issues, we believe technology-based teleophthalmologic approach will be the inevitable method in the near future. Teleophthalmology is an expanding domain that could mitigate resource-incentive aspects of image analysis, nonmydriatic fundus photographs and remote interpretation, which have been used smoothly in both rural and urban settings.^3^ Fast technological advancements make perimeters, fundus cameras, and OCT machines easily accessible with less costs, all of which will be performed in a widespread manner. Monitorization and documentation of findings for future comparisons constitute the personal normative data system. High reproducibility of testing allows each individual to have his or her own normative data and individualize early detection of baseline changes rather than relying on population norms.^37^ Even at the time of this writing, interesting innovations are ongoing with the incorporation of artificial intelligence and deep learning to ophthalmic diagnosis.^38–46^ We believe these methods will help get an effective diagnosis with teleophthalmologic models, and these models will be an ideal implication area for these innovations.

The effective, applicable technology-based eye screening models require not only comprehensive screening devices, but also detailed questionnaire forms to detect personal risk factors effectively. Thus, the questionnaire form was prepared to take the necessary information from participants as fast and as effectively as possible. It included specific questions which might be risk factors for major eye diseases. Dental health and glaucoma were also found to be related in a prospective cohort study of men with glaucoma. Pasquale et al also identified poor oral health and recent tooth loss as risk factors for primary open-angle glaucoma in men.^47^ Somewhat contrary with the findings of Pasquale et al, in our analysis, we did not find any significant increase in risks associated with subjects that did not have a dental examination for more than 2 years.

In addition to the questionnaire form, basic systemic examination findings are also assessed as risk factors for eye diseases. Substantial proportions of participants had newly detected diabetes, hypertension, and conditions that predisposed them to ocular disease.^48,49^ In all groups, only 25% of the participants had a healthy HbA1c level, only the blood pressure of 33% was in normal limits, and only 28% had a normal range of BMI. The percentages of the participants who have healthy HbA1c levels and a normal range of blood pressure were even less in the referred group. Similarly, as seen in Table 5, both self-reported systemic diseases and systemic evaluations that are done by our mobile unit are among the significant risk factors in this group. The systemic risk factors were found more than literature-based anticipations.^50–52^ We believe that our findings show that there is a great need for a comprehensive public health study in New York City. Our study also points to interesting spots for future epidemiological and public health studies.

In Table 5, the effects of all parameters (demographic characteristics as well as systemic and ocular findings) were evaluated using an RRR calculation. This analysis was also complemented with a Bayesian logistic regression as presented in Table 6. As it was a pilot study, the deficiencies were detected—detailed statistical evaluation of the parameters will help improve our models. Different thresholds for risk groups, giving different weights to each technique instead of the either positive rule, modeling combined testing for diagnosis, and priority queuing for reference, shall further be investigated. All these possibilities for future research require a clinically integrated model to see the coherent match between the teleophthalmologic model and clinical diagnosis. These future studies may shed a further light on the clinical significance of our results beyond the statistically significant findings we presented in the current paper.

One major limitation of our study is that it was not designed with a consecutive clinical and comprehensive eye examination. Even though we aimed to understand the true-false-positive-negative diagnosis rates with the follow-up calls, results presented in Table 7 suggest that this was not really feasible. As seen in Table 7, first, the rate of answered phone calls was quite limited. The rate of follow-up examinations done by ophthalmologists was considerably limited. Follow-up visit results were obtained from the participants through phone conversations by using special follow-up questionnaire forms. Therefore, we had to rely on participants’ self-reports about disease confirmation or contradiction. All these factors were the barriers to report accurate true-false positive-negative diagnosis. This would constitute a further motivation to construct a health record integrated teleophthalmologic model. We believe such a potentially integrated system would provide a well-settled diagnostic prediction model and risk analysis with demographics and past medical history.

## CONCLUSION

In conclusion, our study demonstrates the feasibility of real-time, ophthalmology screening in high risk and low socioeconomics minorities of New York City. Additionally, it has the potential to drastically improve access to ophthalmic care while presenting an opportunity to share health information with the community. We obtained important results that are relevant to the population; however, we also believe that our study has room to improve upon the sensitivity and specificity. We plan to perform further research studies that will be guided by this pilot study.

## Figures and Tables

**TABLE 1.** Demographics and Patient Characteristics

Characteristics	Total (n = 957)	Healthy Ocular Findings (n = 499)	Ophthalmology Referred Patients (n = 458)	Glaucoma Suspects (n = 305)	Anatomic Narrow Angle Suspects (n = 136)	Cataract Suspects (n = 124)	AMD Suspects (n = 9)	Retinal Disorders Suspects (n = 29)	Other Ocular Findings (n = 97)

Age, mean ± std	57.72 ± 12.92	54.49 ± 13.01	61.24 ± 11.87	61.6 ± 11.25	59.74 ± 9.61	67.77 ± 10.9	67.89 ± 11.49	62.31 ± 9.55	60.20 ± 12.11
18≤ age ≤44, n (%)	142 (15%)	104 (21%)	38 (8%)	17 (6%)	6 (4%)	3 (2%)	0 (0%)	1 (3%)	12 (12%)
45≤ age ≤54, n (%)	217 (23%)	133 (27%)	84 (18%)	60 (20%)	34 (25%)	12 (10%)	1 (11%)	7 (24%)	16 (16%)
55≤ age ≤64	318 (33%)	136 (27%)	148 (32%)	104 (34%)	53 (39%)	36 (29%)	3 (33%)	9 (31%)	30 (31%)
Age ≥65	280 (29%)	126 (25%)	188 (41%)	124 (41%)	43 (32%)	73 (59%)	5 (56%)	12 (41%)	39 (40%)
Gender, n (%)									
Female	519 (54%)	278 (56%)	241 (%)	162 (53%)	85 (63%)	64 (52%)	7 (78%)	11 (38%)	51 (53%)
Male	438 (46%)	221 (44%)	217 (%)	143 (47%)	51 (38%)	60 (48%)	2 (22%)	18 (62%)	46 (47%)
Ethnicity, n (%)									
African American	300 (31%)	142 (28%)	158 (34%)	107 (35%)	43 (32%)	34 (27%)	4 (44%)	7 (24%)	27 (28%)
Asian	94 (10%)	52 (10%)	42 (9%)	26 (9%)	11 (8%)	12 (10%)	4 (44%)	1 (3%)	10 (10%)
Caucasian	55 (6%)	35 (7%)	20 (4%)	14 (5%)	3 (2%)	14 (11%)	0 (0%)	1 (3%)	3 (3%)
Hispanic	443 (46%)	235 (47%)	208 (45%)	140 (46%)	68 (50%)	59 (48%)	1 (11%)	18 (62%)	46 (47%)
Other	65 (7%)	35 (7%)	28 (6%)	17 (6%)	11 (8%)	5 (4%)	0 (0%)	2 (7%)	9 (9%)
Self-declaration, n (%)									
Insurance	738 (77%)	383 (77%)	355 (78%)	231 (76%)	100 (74%)	21 (72%)	9 (100%)	21 (72%)	86 (89%)
Current smoker	182 (19%)	89 (18%)	93 (20%)	57 (19%)	27 (20%)	23 (19%)	2 (22%)	7 (24%)	17 (18%)
Sleep apnea	93 (10%)	49 (10%)	44 (10%)	26 (9%)	9 (7%)	14 (11%)	1 (11%)	2 (7%)	10 (10%)
Hypertension	383 (40%)	21 (4%)	219 (48%)	145 (48%)	52 (38%)	70 (56%)	1 (11%)	18 (62%)	44 (45%)
Diabetes mellitus	213 (22%)	13 (3%)	119 (26%)	71 (23%)	29 (21%)	33 (27%)	1 (11%)	28 (97%)	31 (32%)
Dyslipidemia	302 (32%)	20 (4%)	163 (36%)	113 (37%)	46 (34%)	45 (36%)	1 (11%)	14 (48%)	32 (33%)
Glaucoma (all)	62 (6%)	12 (2%)	50 (11%)	44 (14%)	10 (7%)	14 (11%)	0 (0%)	6 (21%)	5 (5%)
Cataracts	113 (12%)	41 (8%)	72 (16%)	48 (16%)	12 (9%)	30 (24%)	3 (33%)	14 (48%)	12 (12%)
Macular degeneration	6 (<1%)	3 (<1%)	3 (<1%)	3 (1%)	2 (1%)	1 (1%)	0 (0%)	0 (0%)	1 (1%)
Retinal disorders	18 (2%)	6 (1%)	12 (3%)	9 (3%)	3 (2%)	2 (2%)	0 (0%)	4 (14%)	5 (5%)
Last dental exam									
<1 year	622 (65%)	319 (64%)	303 (66%)	196 (64%)	87 (64%)	75 (60%)	8 (89%)	16 (55%)	66 (68%)
>2 years	213 (22%)	117 (23%)	96 (21%)	70 (23%)	32 (24%)	31 (25%)	1 (11%)	9 (31%)	14 (14%)
>5 years	108 (11%)	54 (11%)	54 (11%)	36 (12%)	16 (12%)	15 (12%)	0 (0%)	3 (10%)	17 (18%)
Never	14 (1%)	9 (2%)	5 (1%)	3 (1%)	1 (<1%)	3 (2%)	0 (0%)	1 (3%)	0 (0%)
Last eye exam									
<1 year	407 (43%)	178 (36%)	200 (44%)	154 (50%)	65 (48%)	19 (66%)	4 (44%)	19 (66%)	54 (56%)
>2 years	317 (33%)	180 (36%)	108 (24%)	98 (32%)	43 (32%)	4 (14%)	2 (22%)	4 (14%)	21 (22%)
>5 years	171 (18%)	97 (19%)	41 (9%)	41 (13%)	22 (16%)	5 (17%)	3 (33%)	5 (17%)	20 (21%)
Never	61 (6%)	43 (9%)	6 (1%)	12 (4%)	6 (4%)	1 (3%)	0 (0%)	1 (3%)	2 (2%)

**TABLE 2.** Comparison of Self-Reported Diseases and Newly Detected Diseases

Condition	Disease Detected by Screening (n = 567)	Self-Reported Within Condition	Newly Identified Disease	Self-Reported Disease, Not Identified by Screening

Ocular conditions (all)	n = 567	n = 129	n = 438	n = 107
Glaucoma (all)	305	44	261	18
Cataract*	124	30	94	83
Macular degeneration	9	0	9	6
Retinal disorders (DRP and the others)	29	4	25	14
Diabetes or prediabetes^†^	433	213	220	24
Hypertension	344	123	221	200

DRP indicates diabetic retinopathy.

* Patients who self-reported a history of cataracts. No differentiation between being preop or postop.

† Prediabetic state was defined as HbA1c 5.7%–6.4% and diabetes was defined as HbA1c ≥ 6.5%.

**TABLE 3.** Results of Vision Testing from the Telemedicine Protocol

Systematic Variables	Total (n = 957)	Healthy Ocular Findings (n = 499)	Ophthalmology Referred Patients (n = 458)	Glaucoma Suspects (n = 305)	Anatomic Narrow-Angle Suspects (n = 136)	Cataract Suspects (n = 124)	AMD Suspects (n = 9)	Retinal Disorders Suspects (n = 29)	Other Ocular Findings (n = 97)

HbA1c, mean ± std	5.96 ± 1.26	5.79 ± 1.03	6.14 ± 1.44	6.15 ± 1.48	5.94 ± 1.18	6.12 ± 1.25	5.74 ± 0.36	8.72 ± 2.14	6.03 ± 1.45
Healthy A1C (Under 5.3), n (%)	243 (25%)	135 (27%)	108 (24%)	66 (22%)	27 (20%)	25 (20%)	1 (11%)	0 (0%)	27 (28%)
Treading towards prediabetes (5.4–5.6), n (%)	279 (29%)	167 (33%)	112 (24%)	86 (28%)	41 (30%)	23 (19%)	4 (44%)	2 (7%)	22 (23%)
Prediabetic state (5.7–6.4), n (%)	289 (30%)	142 (28%)	147 (32%)	92 (30%)	47 (35%)	50 (40%)	4 (44%)	4 (14%)	31 (32%)
Diabetic (≥6.5), n (%)	144 (15%)	54 (11%)	90 (20%)	60 (20%)	21 (15%)	26 (21%)	0 (0%)	23 (79%)	17 (18%)
Systolic BP, mean ± std	128.8 ± 19.66	127.02 ± 18.47	130.74 ± 20.72	130.50 ± 20.13	128.93 ± 20.32	134.97 ± 21.48	125.56 ± 23.67	137.69 ± 24.75	126.48 ± 19.79
Diastolic BP, mean ± std	78.81 ± 12.02	78.39 ± 12.33	79.27 ± 11.67	79.47 ± 11.77	78.10 ± 11.01	78.77 ± 11.35	73.67 ± 12.13	79.76 ± 10.58	76.18 ± 10.32
Low BP, n (%)	10 (1%)	5 (1%)	5 (1%)	3 (1%)	2 (1%)	3 (2%)	0 (0%)	0 (0%)	2 (2%)
Normal BP, (%)	314 (33%)	184 (37%)	131 (29%)	85 (28%)	41 (30%)	26 (21%)	5 (56%)	8 (28%)	35 (36%)
Pre-HT, n (%)	266 (28%)	130 (26%)	136 (30%)	88 (29%)	45 (33%)	44 (35%)	0 (0%)	5 (17%)	32 (33%)
Stage 1 HT, n (%)	115 (12%)	62 (12%)	53 (12%)	40 (13%)	15 (11%)	12 (10%)	2 (22%)	3 (10%)	13 (13%)
Stage 2 HT, n (%)	243 (25%)	115 (23%)	128 (28%)	87 (29%)	33 (24%)	36 (29%)	2 (22%)	11 (38%)	14 (14%)
Hypertensive urgency, n (%)	8 (<1%)	3 (<1%)	5 (1%)	2 (1%)	0 (0%)	3 (2%)	0 (0%)	2 (7%)	1 (1%)
BMI, mean ± std	28.36 ± 5.86	28.64 ± 6.03	28.05 ± 5.66	27.89 ± 5.46	28.16 ± 6.29	28.38 ± 6.68	28.36 ± 8.76	30.58 ± 6.76	27.71 ± 5.71
Normal range (18.5–24.9), n (%)	265 (28%)	127 (25%)	138 (30%)	94 (31%)	39 (29%)	40 (32%)	5 (56%)	4 (14%)	30 (31%)
Elevated (25–29.9), n (%)	393 (42%)	206 (41%)	187 (41%)	123 (40%)	55 (40%)	45 (36%)	1 (11%)	11 (38%)	42 (43%)
High (≥30), n (%)	299 (31%)	166 (33%)	133 (29%)	88 (29%)	42 (31%)	39 (31%)	3 (33%)	14 (48%)	25 (26%)

AMD indicates age-related macular degeneration; BMI, body mass index; BP, blood pressure; HbA1c, hemoglobin A1c; HT, hypertension.

**TABLE 4.** Results of Vision Testing from the Telemedicine Protocol

Ocular Variables	Total (n = 957)	Healthy Ocular Findings (n = 499)	Ophthalmology Referred Patients (n = 458)	Glaucoma Suspects (n = 305)	Anatomic Narrow-Angle Suspects	Cataract Suspects (n = 124)	AMD Suspects (n = 9)	Retinal Disorders Suspects (n = 29)	Other Ocular Findings (n = 97)

VA (logMAR), mean ± std									
Right eye	0.31 ± 0.32	0.26 ± 0.28	0.37 ± 0.35	0.34 ± 0.32	0.29 ± 0.20	0.52 ± 0.47	0.34 ± 0.19	0.67 ± 0.52	0.35 ± 0.36
Left eye	0.35 ± 0.42	0.27 ± 0.29	0.43 ± 0.51	0.41 ± 0.50	0.33 ± 0.40	0.55 ± 0.53	0.36 ± 0.28	0.74 ± 0.73	0.34 ± 0.31
IOP (mm Hg), mean ± std									
Right eye	15.13 ± 4.23	14.46 ± 3.11	15.86 ± 5.09	16.79 ± 5.50	15.65 ± 4.46	15.34 ± 4.34	16.33 ± 3.77	15.10 ± 4.62	13.80 ± 3.45
Left eye	15.27 ± 4.46	14.56 ± 3.16	16.04 ± 5.45	16.89 ± 6.00	15.77 ± 4.33	15.57 ± 4.74	15.89 ± 4.86	15.46 ± 5.31	14.07 ± 3.49
CCT (μm), mean ± std									
Right eye	544.56 ± 40.43	543.31 ± 39.14	545.86 ± 41.73	545.89 ± 40.61	546.45 ± 39.84	544.26 ± 42.61	550.00 ± 36.90	559.31 ± 32.04	548.77 ± 38.17
Left eye	545.19 ± 40.41	544.54 ± 38.96	545.90 ± 41.97	547.07 ± 41.12	547.95 ± 39.50	546.46 ± 46.45	552.00 ± 43.50	545.70 ± 27.19	550.68 ± 34.97
Reader: VCDR, mean ± std									
Right eye	0.37 ± 0.18	0.33 ± 0.14	0.42 ± 0.20	0.46 ± 0.21	0.38 ± 0.18	0.38 ± 0.19	0.40 ± 0.13	0.42 ± 0.20	0.35 ± 0.16
Left eye	0.38 ± 0.18	0.34 ± 0.14	0.42 ± 0.21	0.47 ± 0.22	0.38 ± 0.18	0.37 ± 0.22	0.39 ± 0.10	0.46 ± 0.21	0.36 ± 0.17
OCT: VCDR, mean ± std									
Right eye	0.56 ± 0.20	0.53 ± 0.18	0.58 ± 0.21	0.62 ± 0.21	0.57 ± 0.19	0.56 ± 0.23	0.58 ± 0.19	0.50 ± 0.25	0.52 ± 0.22
Left eye	0.56 ± 0.21	0.54 ± 0.19	0.58 ± 0.22	0.63 ± 0.20	0.58 ± 0.18	0.52 ± 0.26	0.58 ± 0.11	0.54 ± 0.24	0.53 ± 0.23
RNFL (μm), mean ± std									
Right eye	105.17 ± 16.70	109.99 ± 11.61	99.88 ± 19.60	98.82 ± 20.28	105.40 ± 16.79	97.78 ± 20.52	109.56 ± 9.77	97.59 ± 23.18	100.63 ± 20.21
Left eye	104.19 ± 16.94	108.58 ± 11.72	99.30 ± 20.23	97.71 ± 20.51	105.57 ± 16.64	98.56 ± 17.26	112.89 ± 7.37	92.17 ± 25.00	100.03 ± 20.21
FDT-VF (μm), mean ± std									
Right eye, avg misses	1.56 ± 3.23	0.86 ± 2.23	2.32 ± 3.92	1.79 ± 3.32	1.15 ± 2.76	2.38 ± 4.02	2.50 ± 5.90	6.14 ± 6.05	3.79 ± 4.34
Left eye, avg misses	1.69 ± 3.68	0.81 ± 2.31	2.68 ± 4.57	2.12 ± 4.05	1.23 ± 3.08	2.55 ± 4.66	3.13 ± 6.27	5.96 ± 6.43	4.46 ± 5.37

AMD indicates age-related macular degeneration; CCT, central corneal thickness; FDT-VF, frequency doubling technology visual field; IOP, intraocular pressure; OCT, optical coherence tomography; RNFL, retinal nerve fiber layer; VA, visual acuity; VCDR, vertical cup-to-disc ratio.

**TABLE 5.** Results of Relative Risk Ratio from the Telemedicine Protocol

	Healthy Ocular Findings RRR (95%)	*P* value	Ophthalmology Referred Patients RRR (95%)	*P* value	Glaucoma Referred	*P* value	Narrow-Angle Referred	*P* value	Cataract Referred	*P* value

Age (relative to group age 18–44)										
45≤ age ≤54	0.84 (0.72–0.97)	0.02	1.45 (1.05–1.99)	0.02	2.31 (1.41–3.79)	0.00	3.71 (1.60–8.60)	0.00	2.62 (0.75–9.11)	0.13
55≤ age ≤64	0.65 (0.56–0.76)	0.00	1.97 (1.45–2.61)	0.00	3.06 (1.91–4.90)	0.00	4.42 (1.95–10.03)	0.00	5.17 (1.61–16.61)	0.01
Age ≥65	0.54 (0.46–0.65)	0.00	2.24 (1.68–2.98)	0.00	3.30 (2.07–5.26)	0.00	3.24 (1.41–7.44)	0.01	11.76 (3.78–36.62)	0.00
Gender (relative to male)										
Female	1.06 (0.94–1.20)	0.34	0,94 (0.82–1.07)	0.34	0.96 (0.79–1.15)	0.64	1.41 (1.02–1.94)	0.04	0.90 (0.65–1.25)	0.53
Ethnicity (relative to Caucasian)										
Hispanic	0.83 (0.67–1.04)	0.10	1.29 (0.90–1.86)	0.17	1.24 (0.77–1.99)	0.37	2.81 (0.92–8.64)	0.07	0.52 (0.31–0.87)	0.01
African American	0.74 (0.59–0.94)	0.01	1.45 (1.01–2.09)	0.05	1.40 (0.87–2.26)	0.17	2.63 (0.85–8.17)	0.10	0.45 (0.26–0.77)	0.00
Asian	0.87 (0.66–1.14)	0.31	1.23 (0.81–1.86)	0.33	1.09 (0.62–1.90)	0.77	2.15 (0.63–7.36)	0.23	0.50 (0.25–1.00)	0.05
Others	0.85 (0.63–1.14)	0.28	1.27 (0.82–1.97)	0.29	1.09 (0.60–1.98)	0.78	3.10 (0.91–10.56)	0.07	0.30 (0.12–0.79)	0.01
Self-report Conditions										
Presence of insurance	0.98 (0.85–1.13)	0.77	1.02 (0.87–1.20)	0.78	0.93 (0.75–1.15)	0.51	0.84 (0.59–1.20)	0.34	0.92 (0.63–1.35)	0.67
Smoking	0.92 (0.79–1.09)	0.34	1.08 (0.92–1.27)	0.32	0.98 (0.77–1.24)	0.86	1.05 (0.71–1.56)	0.79	0.97 (0.64–1.48)	0.89
Sleep apnea	1.01 (0.83–1.24)	0.91	0.99 (0.79–1.24)	0.91	0.87 (0.62–1.22)	0.41	0.66 (0.35–1.25)	0.20	1.18 (0.71–1.98)	0.52
Personal history of hypertension	0.73 (0.64–0.84)	0.00	1.37 (1.21–1.56)	0.00	1.36 (1.13–1.63)	0.00	0.93 (0.67–1.28)	0.65	1.94 (1.40–2.70)	0.00
Personal history of DM	0.81(0.69–0.96)	0.01	1.23 (1.06–1.41)	0.00	1.06 (0.85–1.32)	0.60	0.95 (0.65–1.39)	0.78	1.27(0.88–1.83)	0.21
Personal history of dyslipidemia	0.84 (0.73–0.96)	0.01	1.19 (1.05–1.37)	0.01	1.28 (1.06–1.54)	0.01	1.11 (0.80–1.54)	0.54	1.24 (0.88–1.74)	0.22
Personal history of glaucoma	0.35 (0.21–0.59)	0.00	1.77 (1.53–2.04)	0.00	2.43 (2.01–2–94)	0.00	1.14 (0.63–2.06)	0.65	1.84 (1.12–3.00)	0.02
Personal history of cataract	0.67 (0.52–0.86)	0.00	1.39 (1.19–1.63)	0.00	1.39 (1.10–1.77)	0.01	0.72 (0.41–1.26)	0.25	2.38 (1.66–3.42)	0.00
Last dental exam (related to less than 1 year)										
Last dental exam >2 years	1.07 (0.92–1.24)	0.35	0.93 (0.78–1.10)	0.37	1.04 (0.83–1.31)	0.77	1.07 (0.74–1.56)	0.71	1.21 (0.82–1.78)	0.34
Last dental exam >5 years	0.97 (0.80–1.20)	0.81	1.03 (0.84–1.26)	0.80	1.06 (0.79–1.41)	0.71	1.06 (0.65–1.73)	0.82	1.15 (0.69–1.93)	0.59
Last dental exam never	1.25 (0.84–1.87)	0.27	0.73 (0.36–1.49)	0.39	0.68 (0.25–1.87)	0.45	0.51 (0.08–3.41)	0.49	1.78 (0.64–4.95)	0.27
Last eye exam (related to last eye exam less than 1 year)										
Last eye exam >2 years	1.30 (1.12–1.50)	0.00	0.77 (0.66–0.89)	0.00	0.82 (0.66–1.00)	0.06	0.85 (0.59–1.21)	0.37	0.55 (0.36–0.82)	0.00
Last eye exam >5 years	1.30 (1.09–1.54)	0.00	0.77 (0.63–0.93)	0.01	0.63 (0.47–0.85)	0.00	0.81 (0.51–1.26)	0.35	0.74 (0.47–1.16)	0.19
Last eye exam never	1.61 (1.32–1.96)	0.00	0.52 (0.35–0.78)	0.00	0.52 (0.30–0.88)	0.01	0.62 (0.28–1.36)	0.23	0.59 (0.27–1.30)	0.19
Presence of regular eye doctor	0.77 (0.67–0.89)	0.00	1.30 (1.14–1.49)	0.00	1.22 (1.00–1.48)	0.05	1.10 (0.79–1.54)	0.58	1.03 (0.72–1.47)	0.89
Reported vision change	0.92 (0.82–1.04)	0.20	1.09 (0.95–1.25)	0.20	0.97 (0.81–1.17)	0.77	1.18 (0.85–1.62)	0.33	1.73 (1.20–2.49)	0.00
Reported eye injury / surgery	0.80 (0.68–0.95)	0.01	1.24 (1.07–1.43)	0.00	1.24 (1.01–1.53)	0.04	1.02 (0.70–1.49)	0.92	1.04 (0.70–1.54)	0.86
Systemic Findings										
BMI (related to normal range 18.5–24.9)										
BMI elevated (25–29.9)	1.09 (0.93–1.28)	0.26	0.91 (0.78–1.07)	0.26	0.88 (0.71–1.10)	0.26	0.95 (0.65–1.39)	0.80	0.76 (0.51–1.13)	0.17
BMI high (≥30)	1.16 (0.99–1.36)	0.07	0.85 (0.72 –1.01)	0.07	0.83 (0.65–1.05)	0.13	0.95 (0.64–1.43)	0.82	0.86 (0.57–1.30)	0.48
HbA1c (related to healthy A1C level <5.3)										
HbA1c: Treading towards prediabetes (5.4–5.6)	1.08 (0.93–1.25)	0.32	0.90 (0.74–1.10)	0.32	1.13 (0.87–1.49)	0.36	1.32 (0.84–2.08)	0.23	0.80 (0.47–1.37)	0.42
HbA1c: Prediabetic state (5.7–6.4)	0.88 (0.25–1.04)	0.14	1.14 (0.96–1.37)	0.14	1.17 (0.90–1.53)	0.24	1.46 (0.94–2.28)	0.09	1.68 (1.07–2.63)	0.02
HbA1c: Diabetic (6.5 and higher)	0.67 (0.53–0.87)	0.00	1.41 (1.16–1.70)	0.00	1.53 (1.16–2.03)	0.00	1.31 (0.77–2.23)	0.32	1.76 (1.06–2.92)	0.03
Blood pressure (related to normal blood pressure)										
Prehypertension	0.84 (0.72–0.98)	0.02	1.23 (1.03–1.47)	0.02	1.23 (0.96–1.57)	0.11	1.29 (0.88–1.92)	0.19	2.00 (1.27–3.16)	0.00
Stage 1 hypertesion	0.92 (0.76–1.12)	0.42	1.11 (0.87–1.40)	0.40	1.29 (0.95–1.76)	0.11	1.00 (0.58–1.74)	0.99	1.26 (0.66–2.42)	0.48
Stage 2 hypertesion	0.81 (0.69–0.95)	0.01	1.27 (1.06–1.51)	0.01	1.33 (1.04–1.70)	0.03	1.04 (0.68–1.60)	0.85	1.79 (1.12–2.89)	0.02
Hypertensive urgency	0.64 (0.26–1.58)	0.33	1.50 (0.86–2.61)	0.15	0.93 (0.28–3.12)	0.90			4.54 (1.73–11.95)	0.00
Low blood pressure (hypoTA)	0.86 (0.46–1.60)	0.63	1.20 (0.64–2.27)	0.57	1.11 (0.42–2.92)	0.83	1.54 (0.43–5.48)	0.51	3.63 (1.32–10.04)	0.01
Ocular Findings (in either eye)										
IOP >21mm Hg	0.27 (0.17–0.41)	0.00	1.99 (1.78–2.22)	0.00	2.70 (2.30–3.18)	0.00	1.37 (0.91–2.08)	0.13	1.89 (1.28–2.78)	0.00
VCDR ≥0.5 according to specialist	0.60 (0.51–0.70)	0.00	1.60 (1.41–1.82)	0.00	2.50 (2.09–3.01)	0.00	1.11 (0.80–1.53)	0.54	1.24 (0.89–1.73)	0.21
VCDR ≥0.7 according to specialist	0.21 (0.11–0.39)	0.00	1.99 (1.78–2.22	0.00	3.08 (2.66–3.56)	0.00	0.92 (0.51–1.68)	0.79	1.72 (1.07–2.75)	0.02
VCDR ≥0.5 according to OCT	0.82 (0.72–0.94)	0.01	1.27 (1.05–1.53)	0.01	1.89 (1.38–2.57)	0.00	1.31 (0.85–2.01)	0.22	1.00 (0.66–1.51)	0.99
VCDR ≥0.7 according to OCT	1.68 (0.58–0.79)	0.00	1.45 (1.27–1.64)	0.00	2.26 (1.89–2.69)	0.00	0.99 (0.71–1.38)	0.94	1.13 (0.80–1.60)	0.48
VF (FDT) abnormality	0.66 (0.58–0.75)	0.00	1.36 (1.28–1.46)	0.00	1.22 (1.10–1.36)	0.00	0.83 (0.66–1.06)	0.13	1.20 (0.98–1.47)	0.07
RNFL <75 μm	0.07 (0.02–0.21)	0.00	2.04 (1.84–2.21)	0.00	2.76 (2.35–3.24)	0.00	1.29 (0.78–2.14)	0.32	2.57 (1.74–3.79)	0.00
RNFL <95 μm	0.44 (0.35–0.54)	0.00	1.88 (1.67–2.12)	0.00	2.46 (2.07–2.93)	0.00	1.06 (0.75–1.50)	0.76	1.85 (1.33–2.57)	0.00
CCT <535 μm	1.06 (0.94–1.20)	0.34	0.94 (0.82–1.07)	0.35	0.91 (0.75–1.09)	0.31	0.78 (0.57–1.07)	0.13	1.07 (0.77–1.49)	0.68
CCT <510 μm	0.98 (0.85–1.14)	0.81	1.02 (0.87–1.19)	0.81	1.07 (0.86–1.33)	0.56	1.08 (0.75–1.56)	0.68	0.98 (0.66–1.46)	0.91

BMI indicates body mass index; CCT, central corneal thickness; FDT, frequency doubling technology; HbA1c, hemoglobin A1c; IOP, intraocular pressure; OCT, optical coherence tomography; RNFL, retinal nerve fiber layer; RRR, relative risk ratios; VA, visual acuity; VF, visual field; VCDR, vertical cup-to-disc ratio.

**TABLE 6.** Bayesian Regression of Ophthalmological Referral (1,2,3) and Glaucoma Referral (4,5,6)

Variables	(1) Oph. Ref.	(2) Oph. Ref.	(3) Oph. Ref.	(4) Glauc. Ref.	(5) Glauc. Ref.	(6) Glauc. Ref.

Age	0.04* (0.01)	0.04* (0.01)	0.04* (0.01)	0.04* (0.01)	0.02* (0.01)	0.03* (0.01)
Hispanic	0.63*** (0.33)	0.71** (0.34)	0.70*** (0.40)	0.53 (0.40)	0.61 (0.41)	0.60 (0.48)
African American	0.97* (0.33)	1.02* (0.35)	1.00** (0.40)	0.82** (0.40)	0.84** (0.42)	0.86*** (0.48)
Asian	0.57 (0.38)	0.60 (0.40)	0.65 (0.46)	0.38 (0.45)	0.42 (0.47)	0.43 (0.53)
Others	0.94** (0.42)	1.00** (0.43)	1.07** (0.48)	0.62 (0.49)	0.68 (0.50)	0.79 (0.56)
HbA1c	0.61* (0.21)	0.51** (0.22)	0.58** (0.26)	0.41*** (0.21)	0.27 (0.22)	0.48*** (0.24)
Treated glaucoma	1.26* (0.32)	1.04* (0.32)	0.66*** (0.37)	1.54* (0.31)	1.30* (0.32)	1.17* (0.35)
IOP >21mm Hg	2.10*	2.04*	2.39*	1.98*	1.92*	2.27^v^
RNFL <95 μm	(0.28)	(0.29) 1.05* (0.18)	(0.35) 0.80* (0.21)	(0.23)	(0.24) 1.12* (0.18)	(0.28) 0.89* (0.20)
VF Abnormal			0.66*			0.15
Constant	−3.76* (0.49)	−3.53* (0.49)	(0.11) −3.96* (0.55)	−3.92* (0.55)	−3.60* (0.54)	(0.10) −3.86* (0.60)
Observations	956	956	784	956	956	784

Robust standard errors in parentheses.

HbA1c indicates hemoglobin A1c; IOP, intraocular pressure; RNFL, retinal nerve fiber layer; VF, visual field.

* *P* < 0.01

** *P* < 0.05

*** *P* < 0.1

**TABLE 7.** Individual Follow-up

Disease, n	Reached, n (%)	Followed Up With Ophthalmologist, n (%)*	Disease Confirmed, n (%)*	Additional Eye Problem Detected, n (%)*	Required Rx, n (%)*

Cataract, n = 124	47 (38%)	39 (83%)	16 (34%)	7 (15%)	3 (6%)
Glaucoma, n = 305	117 (38%)	82 (70%)	50 (42%)	20 (17%)	0 (0%)
Narrow angle, n = 136	41 (30%)	41 (100%)	18 (44%)	25 (61%)	0 (0%)
Retinal disorders, n = 29	14 (48%)	10 (71%)	11 (79%)	6 (43%)	0 (0%)
Macular degeneration, n = 9	0 (0%)	0 (0%)	0 (0%)	NA	NA

NA indicates not applicable; Rx = Prescription for glasses.

* Percentage based on patients reached.
